# Association of the apolipoprotein B/apolipoprotein A1 ratio with 90-day mortality after acute intracerebral hemorrhage: a single-center cohort study

**DOI:** 10.3389/fneur.2026.1898784

**Published:** 2026-10-29

**Authors:** Jun-Meng Huang, Yong-Bo Ma, Shu-Qiang Zhang, Chao-Yi Huang, Rui Feng, You-Dong Wei, Wen-Song Yang

**Affiliations:** 1 Department of Neurology, The First Affiliated Hospital of Chongqing Medical University, Chongqing, China; 2 Department of Radiology, Chongqing University Fuling Hospital, Chongqing, China; 3 Department of Neurology, University-Town Hospital of Chongqing Medical University, Chongqing, China

**Keywords:** apolipoprotein A-I, apolipoprotein B, intracerebral hemorrhage, mortality, prognosis

## Abstract

**Background and purpose:**

Previous studies have linked lipid measures to hematoma expansion and outcomes after intracerebral hemorrhage (ICH), but prognostic evidence for the apolipoprotein B/apolipoprotein A1 (ApoB/ApoA1) ratio remains limited. We examined the associations of acute-phase ApoB/ApoA1 with 90-day mortality and poor functional outcome after primary ICH and evaluated its incremental prognostic information beyond the ICH score.

**Methods:**

We conducted a single-center observational cohort study of adults with primary ICH who underwent baseline CT within 72 h after symptom onset. The primary outcome was 90-day all-cause mortality; the secondary outcome was poor functional outcome at 90 days. Logistic regression examined associations of ApoB/ApoA1 with outcomes in unadjusted and ICH score-adjusted models. Incremental value beyond the ICH score was assessed using discrimination, Brier score, calibration, and bootstrap internal validation.

**Results:**

Among 470 included patients, 73 (15.5%) died and 190 (40.4%) had poor functional outcome at 90 days. Higher ApoB/ApoA1 was associated with lower 90-day mortality after adjustment for the ICH score (OR per 0.1-unit increase, 0.82; 95% CI, 0.71–0.96; *p* = 0.012), but not with poor functional outcome (OR, 0.97; 95% CI, 0.90–1.06; *p* = 0.531). Adding ApoB/ApoA1 to the ICH score yielded a limited increase in discrimination for 90-day mortality (ΔAUC, 0.027; bootstrap 95% CI, 0.008–0.057; likelihood-ratio *p* = 0.008), with little change in Brier score or calibration. No meaningful improvement was observed for poor functional outcome.

**Conclusion:**

Higher acute-phase ApoB/ApoA1 was associated with lower 90-day mortality, but not poor functional outcome, after accounting for the ICH score. ApoB/ApoA1 provided limited incremental prognostic information for 90-day mortality beyond the ICH score. Clinical usefulness remains uncertain, and external validation is needed.

## 1 Introduction

Spontaneous intracerebral hemorrhage (ICH) is among the most devastating stroke subtypes, with high mortality and substantial long-term disability, making early prognostic assessment clinically important (1). Baseline clinical and neuroimaging markers, such as Glasgow Coma Scale (GCS), hematoma volume, intraventricular hemorrhage (IVH), and hemorrhage location, are strongly associated with outcome (2, 3). However, these variables primarily capture baseline hemorrhagic severity. Subsequent prognosis is also influenced by early hematoma evolution (4), treatment-related factors (5), and host biological responses (6). Accordingly, contemporary guidelines emphasize that severity scales such as the ICH score are useful for quantifying overall hemorrhage severity but should not be used as the sole basis for individual prognostication (7). This creates a rationale for evaluating whether readily available biomarkers can provide incremental prognostic information beyond the ICH score.

Among candidate biomarkers, the circulating lipid profile has attracted increasing interest. In contrast to ischemic vascular disease, in which hyperlipidemia is a major risk factor, ICH has shown a different and sometimes paradoxical lipid profile (8). Epidemiologic studies suggest that lower low-density lipoprotein cholesterol (LDL-C), and less consistently higher high-density lipoprotein cholesterol (HDL-C), are associated with greater ICH risk (9, 10). Prospective population-based evidence has also linked low total cholesterol to an increased risk of lobar ICH (11). Clinical trial evidence has also raised concerns about hemorrhagic stroke risk with intensive statin therapy in secondary stroke prevention (12, 13). Among patients with acute ICH, lower LDL-C has been linked to hematoma expansion, early neurological deterioration, and higher short-term mortality (14, 15), whereas findings for HDL-C have been less consistent (16, 17). These post-ICH findings support further evaluation of lipid-related prognostic biomarkers.

Apolipoprotein B (ApoB) and apolipoprotein A1 (ApoA1) are the principal structural proteins of circulating atherogenic and antiatherogenic lipoprotein particles. ApoB-containing lipoproteins include very-low-density lipoprotein, intermediate-density lipoprotein, low-density lipoprotein, and lipoprotein(a) (18), whereas ApoA1 is the major protein component of high-density lipoprotein (19). The ApoB/ApoA1 ratio therefore provides an integrated measure of the balance between ApoB-containing and ApoA1-containing lipoprotein pathways, rather than the concentration of a single cholesterol fraction. From an analytical perspective, apolipoprotein assays are attractive in the acute-care setting because they do not require fasting and are less sensitive to moderately elevated triglyceride levels (20). Experimental studies indicate that cholesterol availability can affect cerebral arterial smooth muscle cell viability and platelet activation (21, 22). These findings suggest possible vascular and hemostatic links between lipid status and hematoma evolution. Pre-ICH lipid-lowering therapy may further influence the measured ratio (23). These features make ApoB/ApoA1 a biologically plausible and analytically practical biomarker to examine in acute ICH.

Direct evidence regarding ApoB/ApoA1 and prognosis after acute ICH remains limited. A prior single-center study suggested that a higher admission ApoB/ApoA1 ratio was associated with worse long-term outcome after ICH, but interpretation was constrained by a relatively small analytic sample, few mortality events, and limited adjustment for confounding (24). More importantly, most prior studies have focused on biomarker-outcome associations rather than asking whether a biomarker improves prognostic assessment beyond an established bedside severity score. It therefore remains unclear whether acute-phase ApoB/ApoA1 retains prognostic information after accounting for baseline hemorrhagic severity, and specifically whether it provides incremental prognostic value beyond the ICH score.

Accordingly, we examined the associations of acute-phase ApoB/ApoA1 with 90-day outcomes and evaluated its incremental prognostic information beyond the ICH score in a consecutive cohort of patients with primary ICH. The primary outcome was 90-day all-cause mortality, and the secondary outcome was poor functional outcome at 90 days.

## 2 Methods

### 2.1 Study design and setting

This was a single-center, consecutive, observational cohort study. Baseline demographic, clinical, laboratory, and imaging data were obtained from inpatient medical records, and outcomes were assessed prospectively at 90 days after symptom onset. The study period spanned September 2017 to June 2022. The study protocol was approved by the Ethics Committee of the First Affiliated Hospital of Chongqing Medical University (approval No. 2017–075). Written informed consent was obtained from patients or their legally authorized representatives, as appropriate.

### 2.2 Study population

Consecutive adult patients (aged ≥18 years) with CT-confirmed primary ICH who underwent baseline CT within 72 h after symptom onset and had in-hospital ApoB and ApoA1 measurements available were screened for eligibility. Patients with primary IVH, secondary ICH, anticoagulant-associated ICH, leukemia-related ICH, multiple intracranial hemorrhages, or thrombolytic drug-related hemorrhage were excluded. For the present analysis, we additionally excluded patients without ApoB and ApoA1 measurements obtained within 24 h after admission, without 90-day follow-up, or without the data required for the prespecified primary models. All 470 included patients had complete data for ApoB/ApoA1, 90-day outcomes, and the covariates used in the primary ICH score-based models. Baseline albumin was available in 464 patients, who formed the albumin-complete subset for sensitivity models 2 and 3.

### 2.3 Baseline data collection and variable definitions

Baseline demographic and clinical data were abstracted from the electronic medical records, including age, sex, smoking and drinking history, vascular risk factors and comorbidities, pre-ICH medications, admission blood pressure, neurological severity at admission assessed by the GCS, and prestroke functional status assessed using the modified Rankin Scale (mRS). Baseline information was obtained from routine clinical assessment, patient or proxy interviews, and was subsequently verified by senior physicians.

Baseline CT images were stored in Digital Imaging and Communications in Medicine (DICOM) format and independently reviewed by a senior neurologist (W.S.Y.) and a neuroradiologist (S.Q.Z.), both blinded to clinical and outcome data, and any discrepancies were resolved by consensus before analysis. Hematoma segmentation and volume quantification were performed using 3D Slicer (version 5.2.2, www.slicer.org), an open-source, semi-automated software platform validated for three-dimensional medical image processing. Hematoma volume was expressed in mL. Hemorrhage location was classified according to the hematoma epicenter on baseline CT as basal ganglia, thalamic, lobar, brainstem, or cerebellar. For calculation of the ICH score, brainstem and cerebellar hemorrhages were classified as infratentorial, and the remaining locations as supratentorial. IVH was defined as present when IVH volume was greater than zero.

Blood samples for lipid testing were analyzed at the Clinical Laboratory Department of the First Affiliated Hospital of Chongqing Medical University. A fully automated biochemical analyzer (Cobas c701; Roche, Basel, Switzerland) with original laboratory reagents was used. Total cholesterol (TC) and triglycerides (TG) were measured using enzymatic colorimetric assays. HDL-C and LDL-C were measured using direct homogeneous enzymatic colorimetric assays. ApoA1 and ApoB were measured using immunoturbidimetric assays. Non-high-density lipoprotein cholesterol (non-HDL-C) was defined as TC minus HDL-C. ApoB/ApoA1 was calculated as ApoB divided by ApoA1.

### 2.4 Follow-up and outcome assessment

Outcome data were collected at 90 days after symptom onset through standardized telephone interviews with patients or their proxies, conducted by trained research staff blinded to baseline characteristics. Functional status at 90 days was assessed using the mRS, where a score of 6 indicates death. The primary outcome was all-cause mortality within 90 days after symptom onset. The secondary outcome was poor functional outcome at 90 days, defined as an mRS score of 4 to 6 (25).

### 2.5 Statistical analysis

Baseline characteristics were summarized overall and across quartiles of ApoB/ApoA1. Continuous variables were presented as medians with interquartile ranges. Categorical variables were presented as counts and percentages. Between-quartile differences were compared using the Kruskal-Wallis test for continuous variables and the chi-square test or Fisher’s exact test, as appropriate, for categorical variables. *p* values were reported for descriptive purposes. The same tests were used to compare available baseline characteristics between included patients and those excluded because of unavailable 90-day mRS data or delayed apolipoprotein measurement. Descriptive statistics and comparisons were based on the available observations for each variable.

Associations of ApoB/ApoA1 with 90-day mortality and poor functional outcome were examined using logistic regression. We used the original ICH score as the base model (3). For each outcome, two prespecified models were fitted. The unadjusted model included ApoB/ApoA1 only. The full model included the ICH score and ApoB/ApoA1.

Potential nonlinearity in the association between ApoB/ApoA1 and each outcome was examined in the prespecified ICH score-adjusted model using restricted cubic splines (RCS), with four knots placed at 5th, 35th, 65th, and 95th percentiles of ApoB/ApoA1 in the analytic sample. Spline-derived ORs were expressed relative to the median ApoB/ApoA1 value in the relevant analytic sample. Overall and nonlinear associations were evaluated using Wald tests.

Sampling uncertainty in the linear ApoB/ApoA1 effect estimates from the primary and sensitivity models were assessed using nonparametric bootstrap resampling with 10,000 replications. For the linear model, percentile-based bootstrap confidence intervals (CIs) for the ApoB/ApoA1 coefficient were obtained from the empirical bootstrap distribution and transformed to ORs per 0.1 unit increase. Two-sided bootstrap *p* values were additionally calculated using studentized (bootstrap-t) method (26). For the spline model, bootstrap refitting was similarly performed and pointwise percentile 95% confidence bands were constructed for the OR curve at each grid point.

To evaluate the incremental prognostic value of ApoB/ApoA1 beyond the ICH score, we compared a reduced model including the ICH score alone with a full model including both the ICH score and ApoB/ApoA1. Apparent discrimination was assessed using the area under the receiver operating characteristic curve (AUC), and the incremental contribution of ApoB/ApoA1 was assessed using the likelihood-ratio test and the difference in apparent AUC (27). The percentile 95% CI for ΔAUC was estimated using 10,000 bootstrap resamples, with both models refitted and evaluated in each resample. Overall prediction performance and calibration were further evaluated using the Brier score, calibration intercept, and calibration slope (28). Smoothed apparent and bootstrap bias-corrected calibration curves were generated for visual assessment of calibration. Internal validation of model performance used bootstrap optimism correction with 2,000 resamples. In each bootstrap resample, the full modeling procedure was repeated, model performance was evaluated in the bootstrap sample and in the original dataset, and the average optimism was subtracted from the apparent performance to obtain optimism-corrected estimates. Bootstrap bias-corrected calibration curves were generated using 2,000 resamples (29).

Exploratory sensitivity analyses were then performed to assess whether the observed associations were robust to the constituent variables of ICH score and to broader baseline confounder adjustment. Four nested sensitivity models were fitted for each outcome. Sensitivity model 0 included ApoB/ApoA1 and the five hemorrhagic severity components, parameterized as continuous age, continuous GCS, log2-transformed hematoma volume, presence of IVH, and infratentorial location. Sensitivity model 1 additionally included sex, systolic blood pressure, premorbid mRS ≥ 2, pre-ICH antiplatelet use, and pre-ICH lipid-lowering therapy. Pre-ICH lipid-lowering therapy was included because of its potential effects on measured apolipoprotein levels (30). Albumin was included in sensitivity analyses to examine whether the ApoB/ApoA1 association was sensitive to additional adjustment for a nonspecific marker of systemic illness and inflammatory status (31). Because baseline albumin was unavailable in six patients, sensitivity model 2 repeated sensitivity model 1 in the subset with complete albumin data to isolate the effect of sample restriction, and sensitivity model 3 further included albumin in that same subset. These sensitivity analyses were exploratory and were intended to evaluate robustness to alternative severity parameterization and broader measured baseline confounding, rather than to replace the prespecified ICH score-based primary analysis.

Component analyses used three ICH score-adjusted logistic regression models for each outcome, with ApoB, ApoA1, or both entered as predictors. Each apolipoprotein concentration was standardized using its mean and standard deviation (SD) in the primary analytic cohort, with ORs reported per 1-SD increase.

Location-specific analyses assessed whether the association between ApoB/ApoA1 and each outcome differed between lobar and non-lobar ICH. To limit model complexity, basal ganglia, thalamic, brainstem, and cerebellar hemorrhages were pooled as non-lobar ICH. The ICH score-adjusted models were extended by adding a binary indicator of lobar ICH and its interaction with ApoB/ApoA1. Interaction estimates were expressed as ratios of ORs for lobar versus non-lobar ICH.

A survivor-restricted analysis examined the association of ApoB/ApoA1 with an mRS score of 4–5 versus 0–3 among patients alive at 90 days, using unadjusted and ICH score-adjusted logistic regression models.

As an exploratory descriptive analysis, pairwise Spearman correlation coefficients were calculated among ApoB, ApoA1, TC, TG, HDL-C, LDL-C, non-HDL-C, ApoB/ApoA1, LDL-C/HDL-C, and non-HDL-C/HDL-C. The correlation structure was visualized using a heatmap to provide descriptive context for the relationship between ApoB/ApoA1 and conventional lipid measures.

All tests were two-sided and a *p* value <0.05 was considered statistically significant. All analyses were performed using R (version 4.2.3; R Foundation for Statistical Computing, Vienna, Austria).

## 3 Results

A total of 623 patients with spontaneous ICH were screened, of whom 41 were excluded because of ineligible hemorrhage types. Of the remaining 582 patients, 77 were excluded because 90-day mRS data were unavailable and 35 because apolipoprotein measurements were obtained outside the prespecified sampling window, leaving 470 patients in the analytic cohort (Figure 1). At 90 days, 73 patients (15.5%) had died and 190 patients (40.4%) had poor functional outcome. The median ApoB/ApoA1 ratio was 0.67 (IQR, 0.53–0.83), the median age was 65 years (IQR, 53–73), 339 patients (72.1%) were male, the median hematoma volume was 9.49 mL (IQR, 4.11–19.87), and the median ICH score was 1 (IQR, 0–1). Baseline albumin was available in 464 patients.

**Figure 1:**
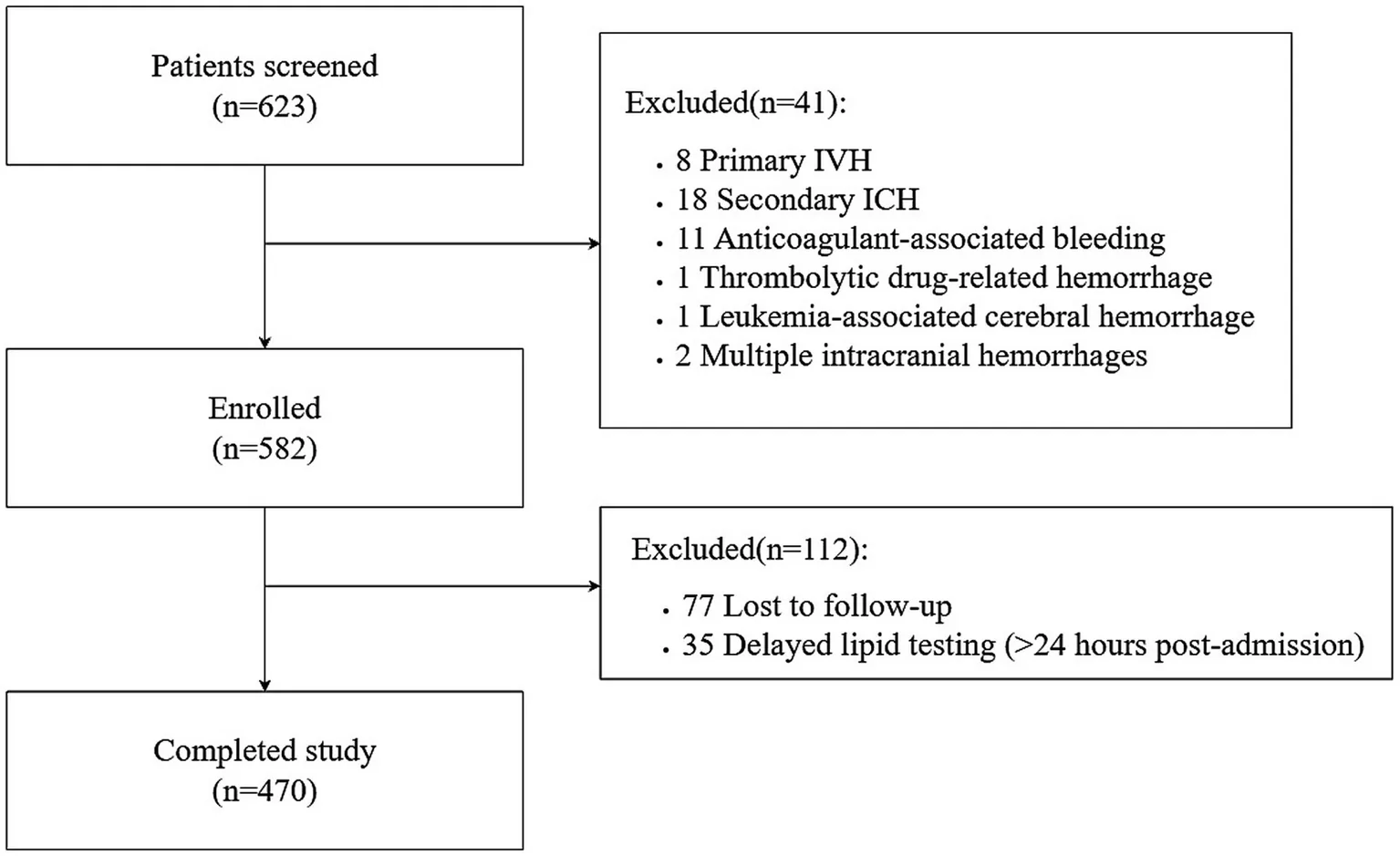
Flow chart of patient selection for the analytic cohort. Patients with primary ICH admitted within 72 h after symptom onset and had ApoB and ApoA1 measurements available in the hospital information system were screened for eligibility. Of 623 screened patients, 41 were excluded because of ineligible hemorrhage types. Among the remaining 582 eligible patients, 112 were excluded because of loss to follow-up at 90 days or ApoB/ApoA1 measurement obtained more than 24 h after admission, leaving 470 patients in the analytic cohort. All 470 patients had complete data for ApoB/ApoA1, 90-day outcomes, and the prespecified covariates. ICH, intracerebral hemorrhage; IVH, intraventricular hemorrhage.

Compared with the 470 included patients, the 112 excluded patients were younger (median, 57 vs. 65 years, *p* = 0.003). A recorded history of hypertension (57.1% vs. 67.7%, *p* = 0.035) and pre-ICH antiplatelet use (0.9% vs. 5.7%, *p* = 0.031) were also less common in the excluded group (Supplementary Table S1).

Baseline characteristics and outcomes across quartiles of ApoB/ApoA1 are presented in Table 1. Participants in higher ApoB/ApoA1 quartiles were younger and more likely to be male. Patients in the lower quartiles had lower admission GCS scores, more frequent IVH, and higher ICH scores. Smoking was more common and albumin levels were modestly higher in the upper quartiles, and pre-ICH antiplatelet and lipid-lowering medication use also differed across quartiles. By contrast, time from symptom onset to initial CT, history of hypertension or diabetes, premorbid disability, hematoma volume, and infratentorial location were broadly similar across quartiles. Mortality at 90 days decreased across increasing quartiles of ApoB/ApoA1, whereas poor functional outcome did not show a clear gradient.

**Table 1:** Baseline characteristics and 90-day outcomes of patients with primary ICH.

Variables	Overall	ApoB/ApoA1				*p* value
		Q1 [0.0667, 0.528]	Q2 (0.528, 0.669]	Q3 (0.669, 0.828]	Q4 (0.828, 2.78]	
	*n* = 470	*n* = 118	*n* = 117	*n* = 117	*n* = 118	
Demographic and clinical characteristics						
Age [median (IQR)]	65.00 [53.00, 73.00]	66.00 [59.00, 74.75]	66.00 [54.00, 74.00]	65.00 [53.00, 71.00]	57.00 [49.00, 69.75]	<0.001
Sex, male (%)	339 (72.1)	68 (57.6)	83 (70.9)	89 (76.1)	99 (83.9)	<0.001
Drinking history (%)	161 (34.3)	37 (31.4)	39 (33.3)	33 (28.2)	52 (44.1)	0.059
Smoking history (%)	222 (47.2)	45 (38.1)	48 (41.0)	59 (50.4)	70 (59.3)	0.004
History of ischemic stroke (%)	40 (8.5)	14 (11.9)	6 (5.1)	7 (6.0)	13 (11.0)	0.149
History of hypertension (%)	318 (67.7)	74 (62.7)	78 (66.7)	82 (70.1)	84 (71.2)	0.501
History of diabetes (%)	76 (16.2)	18 (15.3)	16 (13.7)	20 (17.1)	22 (18.6)	0.749
History of hemorrhagic stroke (%)	33 (7.0)	11 (9.3)	5 (4.3)	10 (8.5)	7 (5.9)	0.401
Coronary heart disease (%)	25 (5.3)	9 (7.6)	6 (5.1)	8 (6.8)	2 (1.7)	0.182
History of chronic renal disease (%)	21 (4.5)	7 (5.9)	6 (5.1)	4 (3.4)	4 (3.4)	0.721
History of chronic hepatic disease (%)	9 (1.9)	2 (1.7)	1 (0.9)	3 (2.6)	3 (2.5)	0.842
Pre-ICH antiplatelet use (%)	27 (5.7)	14 (11.9)	1 (0.9)	6 (5.1)	6 (5.1)	0.004
Pre-ICH lipid-lowering therapy (%)	18 (3.8)	10 (8.5)	0 (0.0)	1 (0.9)	7 (5.9)	<0.001
Premorbid mRS ≥ 2 (%)	19 (4.0)	6 (5.1)	7 (6.0)	2 (1.7)	4 (3.4)	0.344
SBP, mmHg [median (IQR)]	167.00 [150.25, 183.00]	167.00 [153.00, 185.75]	165.00 [147.00, 180.00]	171.00 [154.00, 188.00]	165.00 [149.00, 180.00]	0.168
DBP, mmHg [median (IQR)]	95.00 [84.25, 107.00]	91.50 [84.25, 103.00]	95.00 [82.00, 108.00]	97.00 [85.00, 108.00]	98.50 [86.00, 110.00]	0.071
Surgery (%)	26 (5.5)	4 (3.4)	8 (6.8)	6 (5.1)	8 (6.8)	0.614
Time from onset to initial CT, hours [median (IQR)]	4.00 [2.00, 13.00]	4.00 [2.00, 11.75]	4.00 [2.00, 12.00]	5.00 [2.00, 12.00]	5.50 [2.00, 14.75]	0.721
GCS [median (IQR)]	15.00 [13.00, 15.00]	14.00 [11.00, 15.00]	15.00 [13.00, 15.00]	15.00 [13.00, 15.00]	15.00 [13.00, 15.00]	0.033
Hematoma volume, mL [median (IQR)]	9.49 [4.11, 19.87]	10.67 [4.83, 23.54]	9.24 [4.87, 23.07]	9.49 [3.58, 20.12]	8.71 [3.27, 16.35]	0.149
IVH (%)	116 (24.7)	41 (34.7)	24 (20.5)	27 (23.1)	24 (20.3)	0.031
Hemorrhage location, *n* (%)						0.515
Basal ganglia	228 (48.5)	54 (45.8)	57 (48.7)	61 (52.1)	56 (47.5)	
Thalamic	103 (21.9)	28 (23.7)	23 (19.7)	22 (18.8)	30 (25.4)	
Lobar	86 (18.3)	23 (19.5)	27 (23.1)	20 (17.1)	16 (13.6)	
Brainstem	33 (7.0)	6 (5.1)	6 (5.1)	8 (6.8)	13 (11.0)	
Cerebellar	20 (4.3)	7 (5.9)	4 (3.4)	6 (5.1)	3 (2.5)	
ICH score [median (IQR)]	1.00 [0.00, 1.00]	1.00 [0.00, 2.00]	1.00 [0.00, 1.00]	1.00 [0.00, 1.00]	1.00 [0.00, 1.00]	0.013
Laboratory findings						
TC, mg/dL [median (IQR)]	172.27 [149.75, 199.15]	152.17 [130.51, 173.14]	164.73 [146.56, 192.96]	179.04 [159.32, 203.02]	197.80 [173.24, 218.10]	<0.001
TC, mmol/L	4.45 [3.87, 5.15]	3.94 [3.37, 4.48]	4.26 [3.79, 4.99]	4.63 [4.12, 5.25]	5.12 [4.48, 5.64]	
TG, mg/dL [median (IQR)]	103.18 [70.86, 149.46]	78.38 [56.91, 112.71]	84.14 [64.66, 122.23]	113.37 [85.91, 148.80]	141.71 [110.93, 209.47]	<0.001
TG, mmol/L	1.16 [0.80, 1.69]	0.88 [0.64, 1.27]	0.95 [0.73, 1.38]	1.28 [0.97, 1.68]	1.60 [1.25, 2.37]	
HDL-C, mg/dL [median (IQR)]	48.72 [39.93, 60.71]	59.55 [49.30, 73.76]	52.59 [45.63, 63.81]	46.79 [39.44, 53.36]	39.06 [34.03, 46.40]	<0.001
HDL-C, mmol/L	1.26 [1.03, 1.57]	1.54 [1.27, 1.91]	1.36 [1.18, 1.65]	1.21 [1.02, 1.38]	1.01 [0.88, 1.20]	
LDL-C, mg/dL [median (IQR)]	105.57 [83.91, 132.25]	74.83 [65.45, 90.10]	101.70 [86.62, 124.90]	112.92 [100.93, 133.02]	137.67 [113.40, 155.65]	<0.001
LDL-C, mmol/L	2.73 [2.17, 3.42]	1.94 [1.69, 2.33]	2.63 [2.24, 3.23]	2.92 [2.61, 3.44]	3.56 [2.93, 4.03]	
Non-HDL-C, mg/dL [median (IQR)]	122.78 [98.03, 149.27]	87.97 [74.34, 104.31]	114.46 [96.68, 130.70]	131.48 [116.78, 147.72]	157.58 [136.31, 175.47]	<0.001
Non-HDL-C, mmol/L	3.18 [2.54, 3.86]	2.27 [1.92, 2.70]	2.96 [2.50, 3.38]	3.40 [3.02, 3.82]	4.07 [3.52, 4.54]	
ApoB, mg/dL [median (IQR)]	91.00 [75.25, 110.75]	67.00 [57.00, 78.00]	86.00 [76.00, 95.00]	96.00 [87.00, 108.00]	118.00 [105.00, 127.75]	<0.001
ApoA1, mg/dL [median (IQR)]	135.00 [119.00, 156.00]	158.50 [137.50, 174.75]	141.00 [127.00, 162.00]	130.00 [117.00, 146.00]	117.50 [106.00, 131.00]	<0.001
ApoB/ApoA1 [median (IQR)]	0.67 [0.53, 0.83]	0.43 [0.38, 0.48]	0.60 [0.57, 0.64]	0.75 [0.70, 0.78]	0.96 [0.88, 1.07]	<0.001
*Albumin, g/L [median (IQR)]	41.00 [38.00, 44.00]	40.00 [37.00, 43.00]	41.00 [38.00, 43.00]	42.00 [39.00, 45.00]	42.00 [38.00, 44.00]	0.035
Outcomes						
mRS at 90 days [median (IQR)]	3.00 [1.00, 4.00]	3.00 [2.00, 5.75]	3.00 [2.00, 5.00]	3.00 [1.00, 4.00]	3.00 [1.00, 4.00]	0.030
Mortality at 90 days (%)	73 (15.5)	30 (25.4)	22 (18.8)	14 (12.0)	7 (5.9)	<0.001
mRS 4–6 at 90 days (%)	190 (40.4)	52 (44.1)	55 (47.0)	39 (33.3)	44 (37.3)	0.128

^*^Albumin was available for 464 patients only; albumin values are presented for patients with available data within the ApoB/ApoA1 quartiles defined in the full analytic cohort.

ApoB, apolipoprotein B; ApoA1, apolipoprotein A1; CT, computed tomography; DBP, diastolic blood pressure; GCS, Glasgow Coma Scale; HDL-C, high-density lipoprotein cholesterol; ICH, intracerebral hemorrhage; IVH, intraventricular hemorrhage; LDL-C, low-density lipoprotein cholesterol; mRS, modified Rankin Scale; non-HDL-C, non-high-density lipoprotein cholesterol; SBP, systolic blood pressure; TC, total cholesterol; TG, triglycerides.

In the prespecified ICH score-based models, higher ApoB/ApoA1 was associated with lower 90-day mortality in the unadjusted model (OR per 0.1-unit increase, 0.76; 95% CI, 0.66–0.86; *p* < 0.001). After adjustment for the ICH score, the association remained present (OR, 0.82; 95% CI, 0.71–0.96; *p* = 0.012). Bootstrap analyses yielded similar point estimates and CIs, with a bootstrap-t *p* value of 0.025 for the ICH score-adjusted model (Figure 2). For poor functional outcome, ApoB/ApoA1 showed a modest inverse association in the unadjusted model (OR, 0.92; 95% CI, 0.85–1.00; *p* = 0.044), but this association was no longer evident after adjustment for the ICH score (OR, 0.97; 95% CI, 0.90–1.06; *p* = 0.531). Bootstrap results were directionally consistent with the model-based estimates (Figure 2).

**Figure 2:**
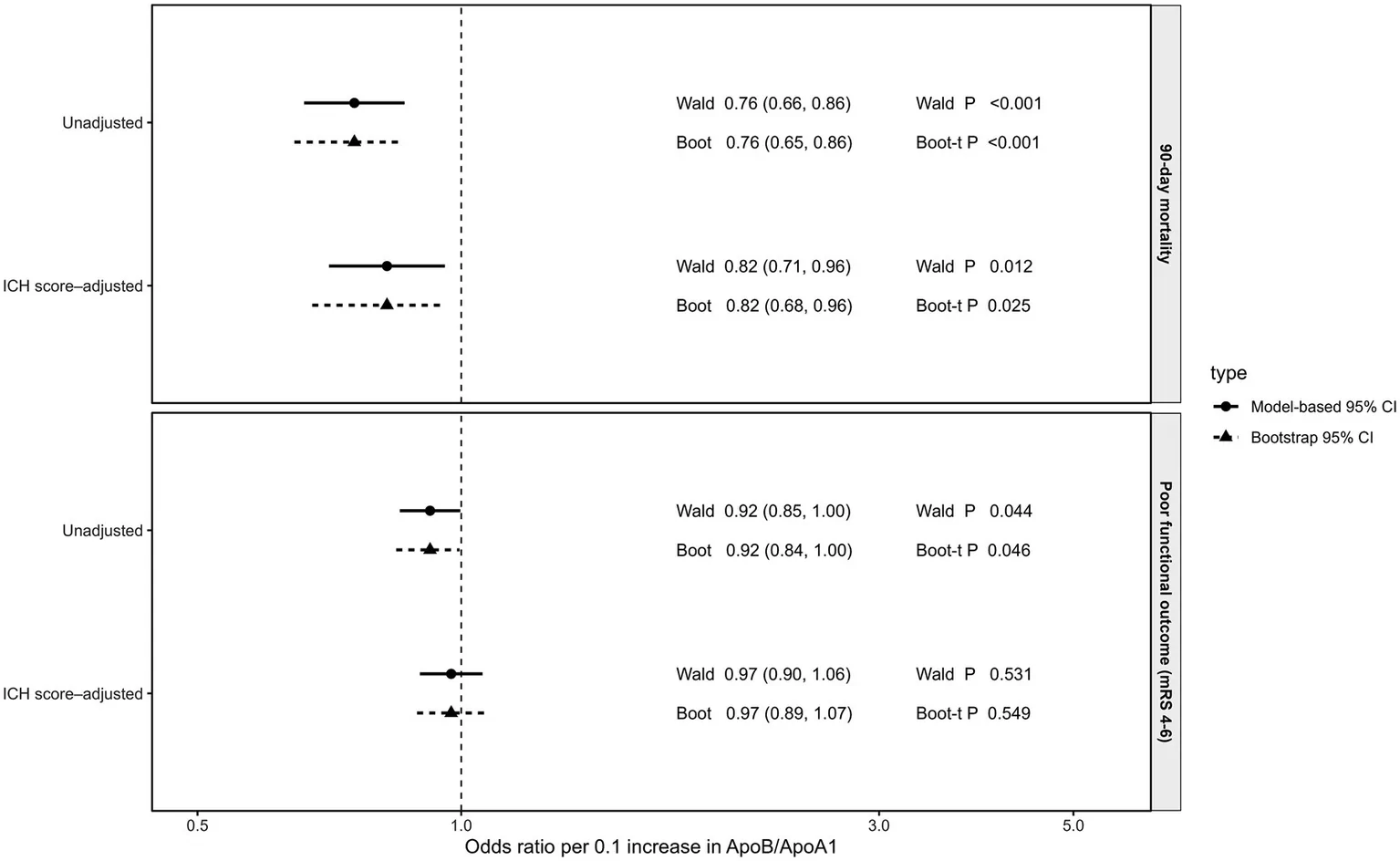
Association of acute-phase ApoB/ApoA1 with 90-day mortality and poor functional outcome. The upper panel shows 90-day mortality, and the lower panel shows poor functional outcome, defined as mRS 4–6 at 90 days. Effect estimates are presented as odds ratios per 0.1-unit increase in ApoB/ApoA1. Two models are shown for each outcome: an unadjusted model and an ICH score-adjusted model. The ICH score was calculated from age, GCS score, hematoma volume, intraventricular hemorrhage, and infratentorial origin according to the original scoring framework. Solid circles and solid lines represent model-based OR and 95% CI; triangles and dashed lines represent bootstrap estimates and 95% CI. *p* values shown on the right are Wald *p* values for the model-based estimates and bootstrap-t *p* values for the bootstrap estimates. ApoA1, apolipoprotein A1; ApoB, apolipoprotein B; CI, confidence interval; ICH, intracerebral hemorrhage; mRS, modified Rankin Scale; OR, odds ratio.

In the ICH score-adjusted RCS model, there was evidence of an overall association between ApoB/ApoA1 and 90-day mortality (P overall = 0.021), but no clear evidence of nonlinearity (P for nonlinearity = 0.094) (Figure 3). Confidence intervals widened at the sparsely populated tails of the ApoB/ApoA1 distribution, limiting interpretation of the curve shape in these regions. The spline curve was directionally consistent with an inverse association across part of the observed range of ApoB/ApoA1, and did not suggest a strong threshold effect. For poor functional outcome, the spline analysis showed no evidence of an overall association (P overall = 0.825) or nonlinearity (P for nonlinearity = 0.776) (Figure 3).

**Figure 3:**
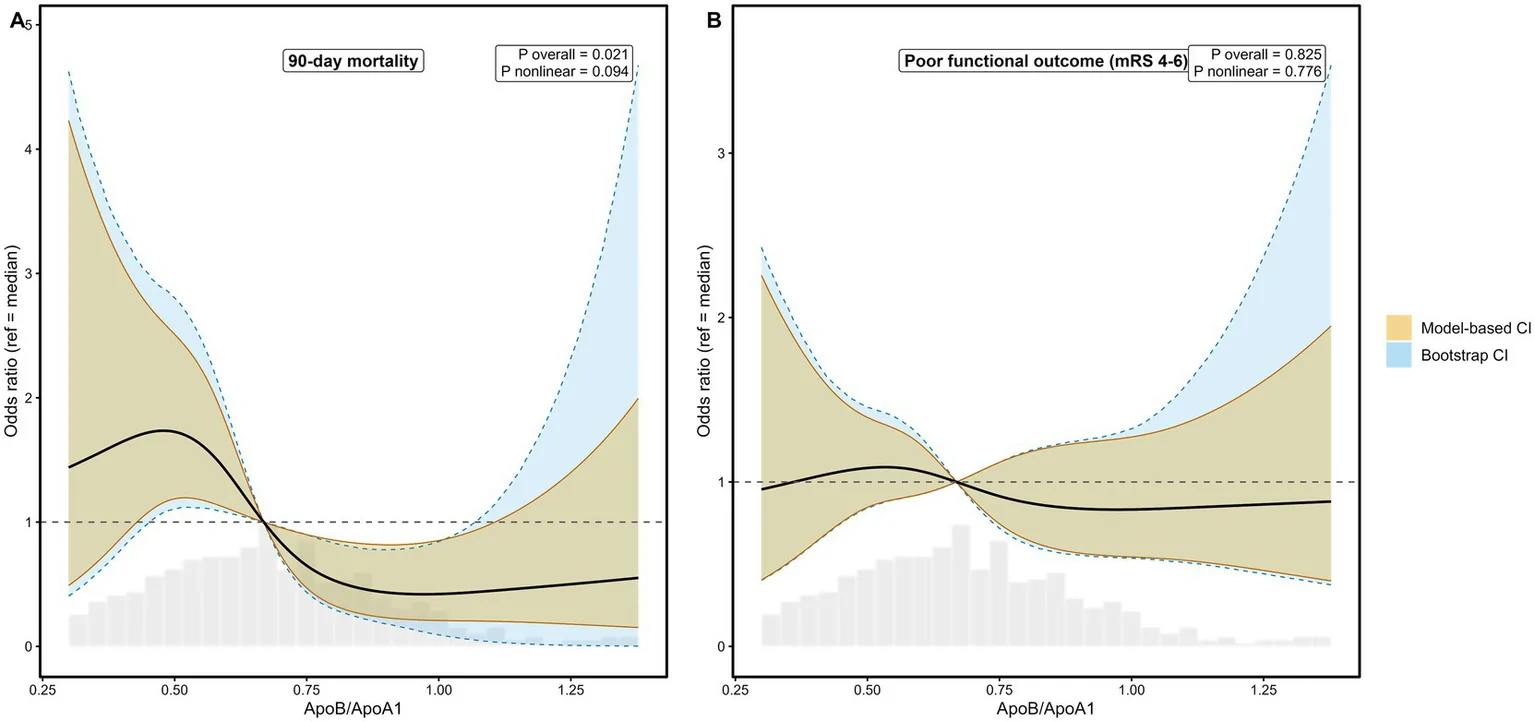
Restricted cubic spline curves for ApoB/ApoA1 and 90-day outcomes in ICH score-adjusted models. Restricted cubic spline plots for ApoB/ApoA1 and 90-day mortality **(A)** and poor functional outcome **(B)** after adjustment for the ICH score. The solid black line indicates the estimated OR relative to the median ApoB/ApoA1. Orange and blue shaded areas represent model-based and bootstrap pointwise 95% CI, respectively. Histograms show the distribution of ApoB/ApoA1. The curves and histograms are displayed between the 1st and 99th percentiles of ApoB/ApoA1, while all 470 patients were retained in model fitting. Confidence intervals widen toward the sparsely populated tails of the ApoB/ApoA1 distribution, so the curve shape in these regions remains uncertain and requires further evaluation in larger cohorts. *p* values indicate the overall and nonlinear associations. CI, confidence interval; ICH, intracerebral hemorrhage; mRS, modified Rankin Scale; OR, odds ratio; RCS, restricted cubic spline.

To evaluate the incremental prognostic value of ApoB/ApoA1 beyond the ICH score, we compared an ICH score-alone model with an ICH score plus ApoB/ApoA1 model. For 90-day mortality, adding ApoB/ApoA1 improved model fit (likelihood-ratio test *p* = 0.008) and yielded a small increase in discrimination (ΔAUC, 0.027; bootstrap 95% CI, 0.008–0.057) (Table 2 and Supplementary Figure S1). The optimism-corrected AUCs were 0.832 for the ICH score-alone model and 0.857 for the model including ApoB/ApoA1. Changes in Brier score were minimal, and calibration remained similar between models, with optimism-corrected intercepts close to 0 and slopes close to 1 (Table 3 and Figure 4).

**Table 2:** Incremental discrimination of ApoB/ApoA1 beyond the ICH score for 90-day mortality and poor functional outcome after ICH.

Outcome	ΔAUC (full-reduced)	Bootstrap 95% CI for ΔAUC	LRT *p* value
90-day mortality	0.027	0.008, 0.057	0.008
Poor functional outcome (mRS 4–6)	0.005	−0.005, 0.025	0.528

ΔAUC represents the difference in apparent AUC between the full model and the reduced model. The reduced model consisted of the ICH score alone, and the full model consisted of the ICH score plus ApoB/ApoA1. Percentile 95% CIs for ΔAUC were obtained using 10,000 nonparametric bootstrap resamples. LRT *p* values were derived from comparison of the nested reduced and full logistic regression models. Poor functional outcome was defined as a mRS score of 4 to 6 at 90 days.

ApoB, apolipoprotein B; ApoA1, apolipoprotein A1; AUC, area under the receiver operating characteristic curve; CI, confidence interval; ICH, intracerebral hemorrhage; LRT, likelihood ratio test; mRS, modified Rankin Scale.

**Table 3:** Apparent and optimism-corrected performance of ICH score-based models.

Outcome	Model	AUC (apparent)	AUC (optimism-corrected)	Brier (apparent)	Brier (optimism-corrected)	Calibration intercept (corrected)	Calibration slope (corrected)
90-day mortality	Reduced model	0.831	0.832	0.090	0.091	0.020	1.005
	Full model	0.858	0.857	0.088	0.089	0.000	0.990
Poor functional outcome	Reduced model	0.709	0.709	0.200	0.201	0.004	1.007
	Full model	0.714	0.708	0.200	0.202	−0.004	0.989

The reduced model included the ICH score alone, and the full model included the ICH score plus ApoB/ApoA1. Optimism-corrected metrics were obtained by bootstrap internal validation using 2,000 resamples. Poor functional outcome was defined as a mRS score of 4 to 6 at 90 days.

ApoB, apolipoprotein B; ApoA1, apolipoprotein A1; AUC indicates area under the receiver operating characteristic curve; ICH, intracerebral hemorrhage; mRS, modified Rankin Scale.

**Figure 4:**
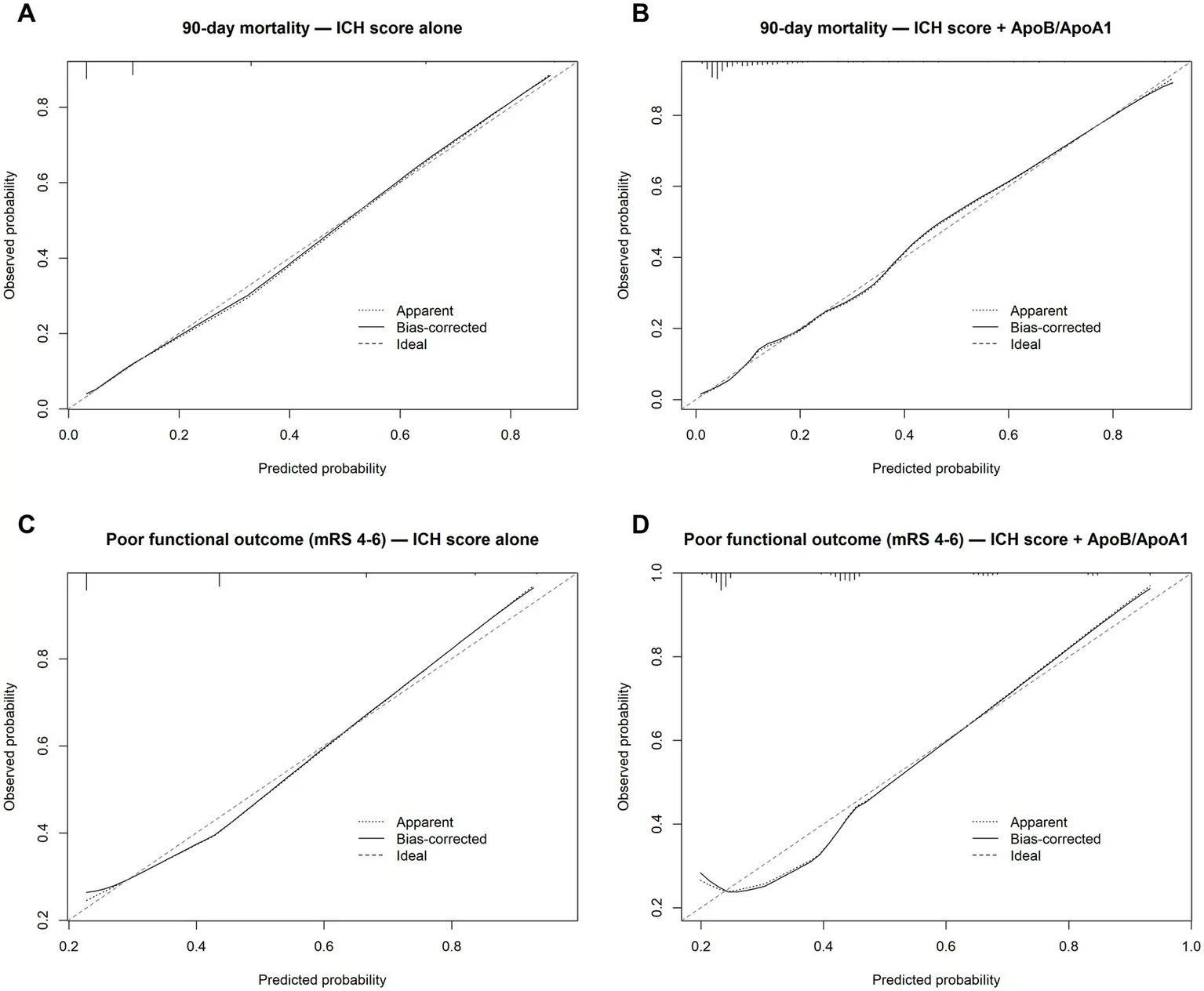
Calibration of ICH score-based models for 90-day mortality and poor functional outcome. Smoothed calibration curves for 90-day mortality are shown for the ICH score alone **(A)** and the ICH score plus ApoB/ApoA1 **(B)**. Corresponding curves for poor functional outcome (mRS 4 -6) are shown for the ICH score alone **(C)** and the ICH score plus ApoB/ApoA1 **(D)**. The dotted line represents the apparent calibration curve, the solid line the bootstrap bias-corrected calibration curve, and the dashed line the ideal line. Calibration curves were internally validated using bootstrap resampling (*n* = 2,000).

For poor functional outcome, adding ApoB/ApoA1 to the ICH score did not improve model fit (likelihood-ratio test *p* = 0.528) and produced no meaningful gain in discrimination (ΔAUC, 0.005; bootstrap 95% CI, −0.005 to 0.025) (Table 2 and Supplementary Figure S2). After bootstrap internal validation, the optimism-corrected AUCs were essentially unchanged between the ICH score-alone and full models (0.709 vs. 0.708), with corresponding optimism-corrected Brier scores of 0.201 and 0.202. Calibration was also similar between models, with intercepts close to 0 and slopes close to 1 (Table 3 and Figure 4). These findings indicate that adding ApoB/ApoA1 did not meaningfully improve overall prediction performance for poor functional outcome.

In exploratory sensitivity analyses, replacing the composite ICH score with its constituent components, the inverse association between higher ApoB/ApoA1 and lower 90-day mortality remained directionally consistent (Supplementary Table S2). In sensitivity model 0, which included ApoB/ApoA1 plus age, GCS, log2-transformed hematoma volume, IVH, and infratentorial location, the OR was 0.85 (95% CI, 0.72–0.99, *p* = 0.039). After additional adjustment for sex, systolic blood pressure, premorbid mRS ≥ 2, pre-ICH antiplatelet use, and pre-ICH lipid-lowering therapy (sensitivity model 1), the estimate was essentially unchanged (OR, 0.85; 95% CI, 0.72–1.00; *p* = 0.045). Restriction to the 464 patients with complete albumin data yielded a similar estimate in sensitivity model 2 (OR, 0.85; 95% CI, 0.72–1.00; *p* = 0.054), and further adjustment for albumin in sensitivity model 3 minimally changed the point estimate (OR, 0.86; 95% CI, 0.73–1.01; *p* = 0.061). Although the point estimates remained directionally similar, neither model-based estimate retained statistical significance at the conventional 0.05 threshold. Across these models, bootstrap estimates were similar, although statistical precision was modestly reduced in the albumin-complete subset. For poor functional outcome, there was no evidence of an association in sensitivity model 0 (OR, 1.03; 95% CI, 0.94–1.12; *p* = 0.552), sensitivity model 1 (OR, 1.04; 95% CI, 0.95–1.14; *p* = 0.395), sensitivity model 2 (OR, 1.04; 95% CI, 0.95–1.14; *p* = 0.378), or sensitivity model 3 (OR, 1.04; 95% CI, 0.95–1.14; *p* = 0.407).

In component analyses, higher ApoB was associated with lower 90-day mortality after adjustment for the ICH score (OR per 1-SD increase, 0.68, 95% CI 0.50–0.92, *p* = 0.014). The point estimate changed little after additional adjustment for ApoA1 (OR, 0.67, 95% CI 0.49–0.91, *p* = 0.011). No clear mortality association was observed for ApoA1 in either the separate or mutually adjusted model. Neither component showed a clear association with poor functional outcome in the separate or mutually adjusted models (Supplementary Table S3).

The location-specific analyses included 470 patients, of whom 86 (18.3%) had lobar ICH and 384 (81.7%) had non-lobar ICH. The mortality ORs derived from the ICH score-adjusted interaction model were 0.83 (95% CI 0.71–0.98, *p* = 0.028) in non-lobar ICH and 0.76 (95% CI 0.50–1.15, *p* = 0.193) in lobar ICH. Interaction analyses provided no clear evidence that the association between ApoB/ApoA1 and either outcome differed by location. The ratios of ORs for lobar versus non-lobar ICH were 0.91 (95% CI 0.58–1.43, P for interaction = 0.676) for 90-day mortality and 0.81 (95% CI 0.60–1.09, P for interaction = 0.113) for poor functional outcome (Supplementary Table S4).

The survivor-restricted analysis included 397 patients alive at 90 days, of whom 117 had an mRS score of 4–5. ApoB/ApoA1 showed no clear association with mRS 4–5 versus 0–3 after adjustment for the ICH score (OR, 1.02, 95% CI 0.94–1.11, *p* = 0.619; Supplementary Table S5).

The correlation structure among ApoB, ApoA1, conventional lipid measures, and lipid-related ratios is shown in Supplementary Figure S2.

## 4 Discussion

In this cohort of patients with primary ICH admitted within 72 h after symptom onset, higher acute-phase ApoB/ApoA1 was associated with lower 90-day mortality after adjustment for the ICH score, whereas no such association was evident for poor functional outcome after the same adjustment. RCS analyses showed an overall association with mortality but no clear evidence of nonlinearity, whereas neither an overall association nor nonlinearity was evident for poor functional outcome. When ApoB/ApoA1 was added to an ICH score-based model, there was a limited improvement in discrimination for 90-day mortality, with little change in overall prediction error or calibration. In exploratory sensitivity analyses replacing the composite ICH score with its individual components, the mortality association remained directionally consistent after broader baseline adjustment. Taken together, these findings suggest that acute-phase ApoB/ApoA1 may provide limited additional prognostic information for short-term survival after ICH beyond the ICH score, but appears less informative for prediction of 90-day functional outcome.

These findings should be interpreted in the context of the limited prior literature on ApoB/ApoA1 in ICH. A previous single-center study reported that a higher admission ApoB/ApoA1 ratio was associated with worse long-term outcome after ICH (24). Several differences may contribute to the discrepancy between that report and the present study, including differences in follow-up duration, exposure modeling, adjustment strategy, and case mix. At the same time, our findings are not entirely isolated from the broader ICH lipid literature, in which lower LDL-C (14), lower non-HDL-C (32), or lower LDL-C/HDL-C ratios (33), have often been linked to greater hemorrhagic severity, hematoma expansion, or worse short-term outcomes. Taken together, these data suggest that the prognostic significance of acute-phase lipid-related markers in ICH may be context dependent and sensitive to outcome definition, follow-up duration, and the extent to which acute severity is captured in the underlying prognostic framework.

The component analyses showed an inverse association between ApoB and 90-day mortality, with little change in the point estimate after additional adjustment for ApoA1. No clear mortality association was observed for ApoA1. This pattern suggests that ApoB may contribute to the inverse association observed for the ratio.

Several explanations may underlie both the inverse association between ApoB/ApoA1 and 90-day mortality and the additional prognostic information it provides beyond the ICH score. First, a lower acute-phase ApoB/ApoA1 ratio may reflect poorer overall host condition and reduced physiologic reserve. Patients in the lower ApoB/ApoA1 quartiles were older and had slightly lower albumin concentrations. However, albumin is influenced by inflammatory responses and is not a specific measure of nutritional status. Within the albumin-complete subset, additional adjustment for albumin changed the point estimate for the mortality association only slightly. The role of physiologic reserve remains uncertain, and albumin adjustment does not exclude residual confounding by other aspects of general health. Second, the observed association may be linked to pathways related to hemorrhagic severity. One possibility is that low ApoB/ApoA1 partly reflects an acute-phase response to more severe hemorrhagic injury. Severe ICH is accompanied by sympathetic activation, catecholamine release (34), and systemic inflammatory responses (35), all of which may alter circulating lipoprotein concentrations during the acute phase (36). Another possibility is that a lower ApoB/ApoA1 ratio marks a premorbid lipid milieu associated with impaired vascular or hemostatic stability, thereby predisposing to a larger initial hemorrhage or hematoma expansion and, in turn, higher short-term mortality (14, 15). The attenuation of the mortality association after accounting for the ICH score is compatible with either or both of these interpretations, but our data cannot distinguish between them. We lacked premorbid lipid measurements, serial lipid profiles, inflammatory markers, and serial neuroimaging, and therefore cannot determine whether the observed pattern primarily reflected baseline host biology, acute metabolic remodeling after severe ICH, or both. Therefore, these mechanistic interpretations should be viewed as hypothesis-generating. Some ICH score components may reflect processes that also influence acute-phase ApoB/ApoA1 or capture aspects of hemorrhage severity that may mediate the relationship between premorbid lipid status and mortality. ICH score adjustment was used to assess incremental prognostic information, and the adjusted association does not establish an independent causal biological effect of ApoB/ApoA1.

Pre-ICH lipid-lowering therapy was included in the expanded sensitivity models because of its potential effects on measured apolipoprotein levels. In SPARCL, hemorrhagic stroke was more frequent with atorvastatin 80 mg/day than with placebo in patients with a recent stroke or transient ischemic attack (12, 13). Observational studies examining prognosis after ICH have reported inconsistent associations between pre-ICH statin use and mortality or functional outcome (37, 38). In our cohort, pre-ICH lipid-lowering therapy was recorded in only 18 of 470 patients (3.8%), limiting treatment-specific assessment. Residual treatment-related confounding cannot be excluded, and the observed ApoB/ApoA1 association should not be interpreted as a treatment effect.

An important observation in this study is the divergence between mortality and functional outcome. Although ApoB/ApoA1 retained an inverse association with 90-day mortality after adjustment for the ICH score, no such association was seen for poor functional outcome, and no meaningful incremental prognostic value was observed for that endpoint. The exploratory analysis among 90-day survivors likewise showed no clear association between ApoB/ApoA1 and mRS 4–5 versus 0–3. One possible explanation for this pattern is that acute-phase ApoB/ApoA1 partly reflects physiological vulnerability relevant to early fatal deterioration. Death after ICH is an absorbing outcome, whereas 90-day disability represents a later functional snapshot influenced by the combined effects of initial injury, early secondary injury, hospital course, and early recovery (39–42). In addition, the use of mRS 4–6 may have diluted a weak mortality-related signal by combining death with severe disability among survivors, whose 90-day status is shaped by a broader set of post-acute factors. However, the 90-day all-cause mortality endpoint does not distinguish early from later deaths, and the present analyses did not test this explanation. The survivor-restricted findings may be affected by selection bias. The RCS analyses provide further context. The ICH score-adjusted RCS analysis showed no clear evidence of nonlinearity for mortality. Confidence intervals widened substantially at the sparsely populated tails of the ApoB/ApoA1 distribution, limiting interpretation of the curve shape in these regions.

This study has several strengths. First, hematoma volume was quantified using semi-automated volumetric segmentation, which likely improved the precision of neuroimaging measurement (43). Second, the analytic framework moved beyond conventional association testing by evaluating whether ApoB/ApoA1 added prognostic information beyond a clinically familiar bedside severity score, while also incorporating spline modeling, bootstrap assessment of estimate stability, and formal evaluation of discrimination, calibration, and internal validation.

Several limitations warrant acknowledgment. First, this was an observational single-center study, and causality cannot be inferred and generalizability is limited. Second, restriction to patients with early apolipoprotein measurements and complete model data may have introduced selection bias, particularly because included and excluded patients differed in several baseline characteristics. Third, ApoB and ApoA1 were measured only once during the acute hospitalization. A single early value cannot distinguish premorbid lipid biology from acute-phase changes after ICH. Fourth, we lacked serial neuroimaging and detailed data on hematoma expansion, edema progression, in-hospital complications, treatments, and cause-specific mortality, limiting mechanistic interpretation. Fifth, only 14 deaths occurred among the 86 patients with lobar ICH, limiting precision in assessing the association between ApoB/ApoA1 and mortality in this subgroup. Non-lobar sites were pooled to limit model complexity, so differences in these associations among non-lobar sites were not assessed. Finally, the prognostic performance estimates and the incremental value of ApoB/ApoA1 were internally assessed only and therefore require external validation. Future multicenter prospective studies incorporating premorbid lipid data, serial apolipoprotein measurements, and longitudinal neuroimaging are needed to clarify mechanisms and determine clinical value.

In conclusion, higher acute-phase ApoB/ApoA1 was associated with lower 90-day mortality after ICH, but was not associated with poor functional outcome after adjustment for the ICH score. Adding ApoB/ApoA1 to the ICH score yielded a limited improvement in discrimination for 90-day mortality, with little change in overall prediction error or calibration. The clinical usefulness of this incremental information remains uncertain, and external validation is required.

## Data Availability

The raw data supporting the conclusions of this article will be made available by the authors, without undue reservation.
