# Supplementary material for: Association of the apolipoprotein B/apolipoprotein A1 ratio with 90-day mortality after acute intracerebral hemorrhage: a single-center cohort study
Source: Front Neurol. 2026 Oct 29;17:1898784. doi: 10.3389/fneur.2026.1898784 (PMC13754555; doi:10.3389/fneur.2026.1898784)
Supplement: Supplementary file 1 [file Data_Sheet_1.pdf]

Supplementary Figure S1. ROC curves for ICH Score-Based Reduced and Full Models for 90-Day Mortality and Poor Functional Outcome

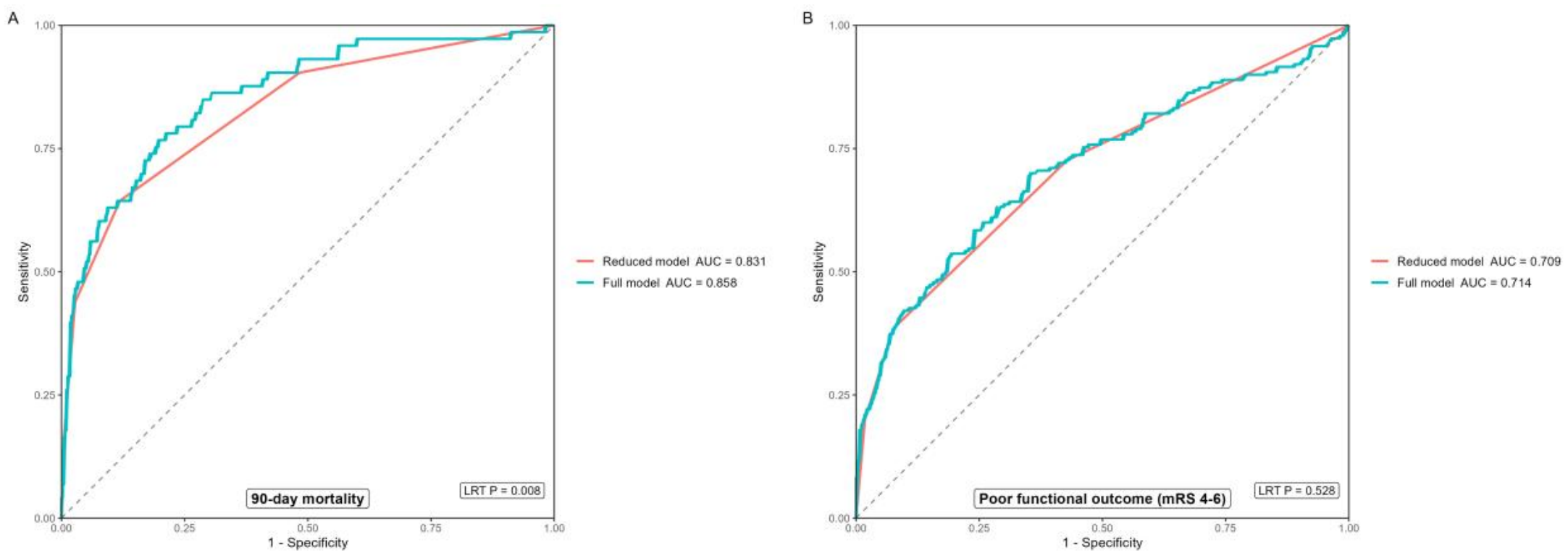

ROC curves for 90-day mortality (A) and poor functional outcome (B) comparing the ICH score alone with the ICH score plus ApoB/ApoA1. AUC values are displayed for each model, and LRT P values indicate comparison of the nested reduced and full models.

Abbreviations: AUC, area under the curve; ICH, intracerebral hemorrhage; LRT, likelihood-ratio test; mRS, modified Rankin Scale; ROC, receiver operating characteristic

Supplementary Figure S2. Spearman correlation heatmap of apolipoproteins, conventional lipid measures, and lipid-related ratios in the analytic cohort

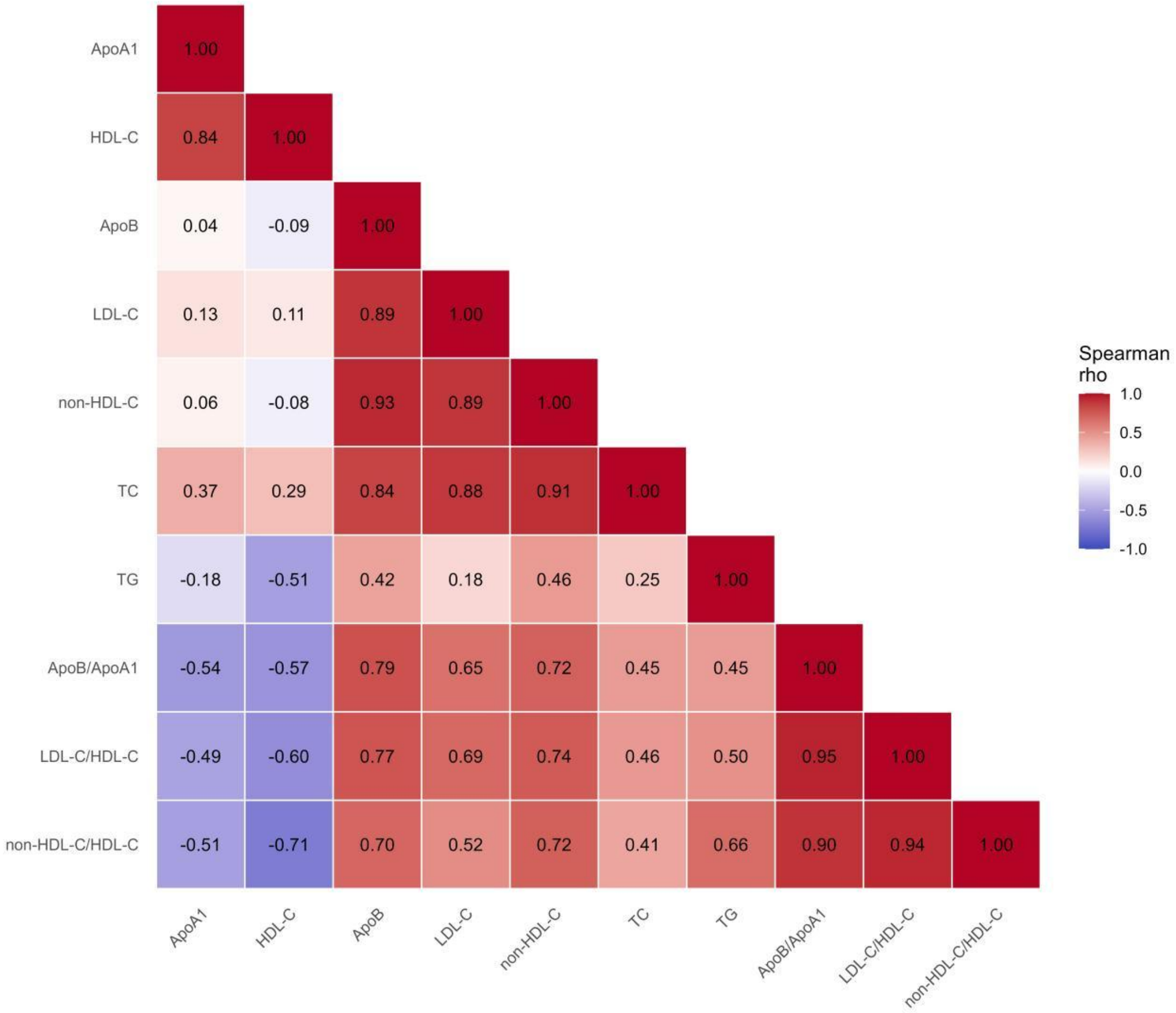

The value in each tile represents the Spearman correlation coefficient (ρ). Color intensity indicates the direction and magnitude of correlation, with red indicating positive correlation and blue indicating negative correlation.

Abbreviations: ApoA1, apolipoprotein A1; ApoB, apolipoprotein B; TC, total cholesterol; TG, triglycerides; HDL-C, high-density lipoprotein cholesterol; LDL-C, low-density lipoprotein cholesterol; non-HDL-C, non-high-density lipoprotein cholesterol

Supplementary Table S1. Available baseline characteristics of patients included in and excluded from the analytic cohort			
Characteristic	Included patients (n=470)	Excluded patients (n=112)	P value
Demographic and clinical characteristics			
Age, years	65.0 [53.0, 73.0]	57.0 [49.5, 68.5]	0.003
Sex, male	339/470 (72.1%)	73/112 (65.2%)	0.146
Premorbid mRS ≥2	19/470 (4.0%)	4/112 (3.6%)	1.000
GCS score	15 [13, 15]	15 [13, 15]	0.885
Systolic blood pressure, mmHg	167.0 [150.2, 183.0]	162.5 [145.2, 180.5]	0.174
Diastolic blood pressure, mmHg	95.0 [84.2, 107.0]	93.0 [80.2, 104.0]	0.246
Medical history and pre-ICH medication			
Smoking history	222/470 (47.2%)	53/112 (47.3%)	0.987
Drinking history	161/470 (34.3%)	33/112 (29.5%)	0.334
History of hypertension	318/470 (67.7%)	64/112 (57.1%)	0.035
History of diabetes	76/470 (16.2%)	12/112 (10.7%)	0.148
History of atrial fibrillation	4/470 (0.9%)	1/112 (0.9%)	1.000
History of ischemic stroke	40/470 (8.5%)	4/112 (3.6%)	0.076
History of hemorrhagic stroke	33/470 (7.0%)	9/112 (8.0%)	0.709
Coronary heart disease	25/470 (5.3%)	2/112 (1.8%)	0.110
History of chronic renal disease	21/470 (4.5%)	4/112 (3.6%)	0.800
History of chronic hepatic disease	9/470 (1.9%)	2/112 (1.8%)	1.000
Pre-ICH antiplatelet use	27/470 (5.7%)	1/112 (0.9%)	0.031
Pre-ICH lipid-lowering therapy	18/470 (3.8%)	1/112 (0.9%)	0.145
Pre-ICH antihypertensive therapy	142/470 (30.2%)	27/112 (24.1%)	0.201
Albumin, g/L	41.0 [38.0, 44.0], n=464	41.0 [38.0, 44.0]	0.966

The comparison included 470 patients in the analytic cohort and 112 excluded patients. Analyses used the available data for each variable without imputation, and variable-specific denominators are reported. Albumin values were available for 93 of the 112 excluded patients.

Abbreviations: CT, computed tomography; GCS, Glasgow Coma Scale; ICH, intracerebral hemorrhage; mRS, modified Rankin Scale.

Supplementary Table S2. Exploratory sensitivity analyses of the association between ApoB/ApoA1 and 90-day outcomes after alternative parameterization of hemorrhagic severity and broader baseline adjustment

Sensitivity model	Analytic sample, n	Events, n (%)	OR(95% CI)	P value
90-day mortality				
Model 0: severity components only	470	73(15.5)	0.85(0.72-0.99)	0.039
Model 1:model 0 + broader baseline	470	73(15.5)	0.85(0.72-1.00)	0.045
Model 2: model 1 in albumin-complete	464	73(15.7)	0.85(0.72-1.00)	0.054
Model 3: model 2 + albumin	464	73(15.7)	0.86(0.73-1.01)	0.061
Poor functional outcome at 90 days (mRS 4–6)				
Model 0: severity components only	470	190(40.4)	1.03(0.94-1.12)	0.552
Model 1: model 0 + broader baseline	470	190(40.4)	1.04(0.95-1.14)	0.395
Model 2: model 1 in albumin-complete	464	188(40.5)	1.04(0.95-1.14)	0.378
Model 3: model 2 + albumin	464	188(40.5)	1.04(0.95-1.14)	0.407

Odds ratios are reported per 0.1-unit increase in ApoB/ApoA1. Sensitivity model 0 replaced the composite ICH score with its constituent severity components (age, GCS, log2-transformed hematoma volume, IVH, and infratentorial location). Sensitivity model 1 additionally adjusted for sex, systolic blood pressure, premorbid mRS ⩾2, pre-ICH antiplatelet use, and pre-ICH lipid-lowering therapy. Sensitivity model 2 repeated sensitivity model 1 in the subset with complete albumin data to isolate the effect of sample restriction. Sensitivity model 3 further included albumin in the same subset.

Abbreviations: ApoB, apolipoprotein B; ApoA1, apolipoprotein A1; CI, confidence interval; OR, odds ratio; GCS, Glasgow Coma Scale; ICH, intracerebral hemorrhage; IVH, intraventricular hemorrhage; mRS, modified Rankin Scale.

Supplementary Table S3. Associations of ApoB and ApoA1 with 90-day outcomes in separate and mutually adjusted models

Model	Component	OR per 1-SD increase (95% CI)	P value
90-day mortality (n = 470, 73 deaths)			
ICH score + ApoB	ApoB	0.68 (0.50 - 0.92)	0.014
ICH score + ApoA1	ApoA1	1.03 (0.78 - 1.37)	0.828
ICH score + ApoB + ApoA1	ApoB	0.67 (0.49 - 0.91)	0.011
ICH score + ApoB + ApoA1	ApoA1	1.10 (0.82 - 1.47)	0.527
Poor functional outcome at 90 days (mRS 4–6) (n = 470, 190 events)			
ICH score + ApoB	ApoB	0.96 (0.79 - 1.18)	0.709
ICH score + ApoA1	ApoA1	1.04 (0.85 - 1.27)	0.717
ICH score + ApoB + ApoA1	ApoB	0.96 (0.78 - 1.18)	0.691
ICH score + ApoB + ApoA1	ApoA1	1.04 (0.85 - 1.28)	0.698

Odds ratios are reported per 1-SD increase in each apolipoprotein. ApoB and ApoA1 were standardized using their respective means and SDs in the common 470-patient analytic cohort. One SD corresponded to 25.29 mg/dL for ApoB and 29.21 mg/dL for ApoA1. All models included the ICH score. In the mutually adjusted model, ApoB and ApoA1 were included simultaneously. Poor functional outcome was defined as an mRS score of 4–6 at 90 days. The 95% CIs and two-sided P values were calculated using the Wald method. These analyses were exploratory.

Abbreviations: ApoB, apolipoprotein B; ApoA1, apolipoprotein A1; CI, confidence interval; ICH, intracerebral hemorrhage; mRS, modified Rankin Scale; OR, odds ratio; SD, standard deviation.

Supplementary Table S4. Exploratory location-specific associations of ApoB/ApoA1 with 90-day outcomes

Location-specific associations derived from the interaction model					
Outcome	Location	n	Events	OR (95% CI)	P value
90-day mortality	Non-lobar	384	59	0.83 (0.71–0.98)	0.028
90-day mortality	Lobar	86	14	0.76 (0.50–1.15)	0.193
Poor functional outcome	Non-lobar	384	163	1.01 (0.92–1.11)	0.892
Poor functional outcome	Lobar	86	27	0.81 (0.61–1.08)	0.146

Interaction estimates comparing the location-specific associations		
Outcome	Ratio of ORs, lobar/non-lobar (95% CI)	P for interaction
90-day mortality	0.91 (0.58–1.43)	0.676
Poor functional outcome	0.81 (0.60–1.09)	0.113

Notes. All analyses included the same 470 patients, with 73 deaths and 190 poor functional outcomes at 90 days. Poor functional outcome was defined as an mRS score of 4–6. Non-lobar ICH included basal ganglia, thalamic, brainstem, and cerebellar hemorrhages.

For each outcome, the logistic regression model included the ICH score as a continuous covariate, continuous ApoB/ApoA1, a binary indicator of lobar ICH, and an ApoB/ApoA1-by-location interaction term.

Abbreviations: ApoB, apolipoprotein B; ApoA1, apolipoprotein A1; CI, confidence interval; ICH, intracerebral hemorrhage; mRS, modified Rankin Scale; OR, odds ratio.

Supplementary Table S5. Association of ApoB/ApoA1 with functional status among 90-day survivors

Model	OR (95% CI)	P value
Unadjusted	1.00 (0.92 to 1.09)	0.915
ICH score-adjusted	1.02 (0.94 to 1.11)	0.619

Notes: Both models included the same 397 patients alive at 90 days from the original 470-patient analytic cohort. The outcome was an mRS score of 4–5 (117 patients), with mRS 0–3 as the reference category (280 patients). The adjusted model additionally included the ICH score as a continuous variable. The 95% CIs and two-sided P values were calculated using the Wald method. Odds ratios are reported per 0.1-unit increase in ApoB/ApoA1.

Abbreviations: ApoB, apolipoprotein B; ApoA1, apolipoprotein A1; CI, confidence interval; ICH, intracerebral hemorrhage; mRS, modified Rankin Scale; OR, odds ratio.
